# Assessment of Cavitation Erosion in a Water-Jet Pump Based on the Erosive Power Method

**DOI:** 10.1155/2021/5394782

**Published:** 2021-12-16

**Authors:** Ning Qiu, Han Zhu, Yun Long, Jinqing Zhong, Rongsheng Zhu, Suhuan Wu

**Affiliations:** ^1^National Research Center of Pumps, Jiangsu University, Zhenjiang, 212013 Jiangsu, China; ^2^Shanghai Bondpoly Engineering Material & Technology Co. Ltd., Shanghai 201601, China

## Abstract

Cavitation affects the performance of water-jet pumps. Cavitation erosion will appear on the surface of the blade under long-duration cavitation conditions. The cavitation evolution under specific working conditions was simulated and analyzed. The erosive power method based on the theory of macroscopic cavitation was used to predict cavitation erosion. The result shows that the head of the water-jet pump calculated using the DCM-SST turbulence model is 12.48 m. The simulation error of the rated head is 3.8%. The cavitation structure of tip leakage vortex was better captured. With the decrease of the net positive suction head, the position where the severe cavitation appears in the impeller domain gradually moves from the tip to the root. The erosion region obtained by the cavitation simulation based on the erosive power method is similar to the practical erosion profile in engineering. As the net positive suction head decreases, the erodible area becomes larger, and the erosion intensity increases.

## 1. Introduction

Compared with traditional propellers, water-jet pumps have the advantages of high propulsion efficiency, good manoeuvrability, and low vibration. It is widely used in the propulsion of high-speed ships. But the performance of the water-jet pump will be affected by the cavitation problem [1–5]. The cavitation plays an important role in the design and operation of hydraulic machinery, and it causes degradation, noise, vibration, and erosion [6, 7]. Cavitation erosion will appear on the surface of the blades when the pump is operated under cavitation conditions for a long time. It will not only affect the reliability of the overall system but also cause high maintenance costs [8–11].

Many scholars have studied the cavitation structure in water-jet pumps and try to explain the effect of cavitation on the performance of water-jet pumps through experiments and simulations. Park et al. [1] conducted experimental research based on PIV technology. The results show that the flow separation phenomenon is easy to occur at the lips of the flow channel when the inflow velocity decreases. Tan et al. [12] observed the formation of perpendicular cavitation vortex (PCV) in the impeller of the water-jet pump by experimental method. The shedding of PCV can cause the head to drop sharply. Motley et al. [13] used high-speed photography to observe the evolution of cavitation on the impeller of a water-jet pump. Cavitation first appeared in the tip clearance of the impeller. Long et al. [14, 15] captured the cavitation structure on the impeller of the water-jet pump at inception cavitation. Wu et al. [16] used the particle image velocity measurement method to study the turbulence structure of the tip leakage vortex of an axial water-jet pump.

Lindau et al. [17] simulated cavitation in the water-jet pump and found that cavitation would cause a sudden drop in thrust and torque. Katz's research shows that the axial shear vortex structure has an impact on the development of cavitation in the separation zone [18]. The numerical result of Guo et al. [19] showed that the pressure pulsation amplitude of the monitors near the tip increases with the extent of cavitation. Huang et al. [20–23] analyzed the cavitation and vortex structures in water-jet pumps. The results show that the evolution of cavitation has aggravated the generation of vortex and flow instability in pumps. When cavitation occurs, the vortex expansion and baroclinic torque appear as violent fluctuation. Xu et al. [24] found that the viscous dissipation term has a larger magnitude at the tip clearance of water-jet pumps.

Cavitation erosion is a hot research currently [25]. Traditionally, cavitation erosion risk is assessed by experimental methods [26–30]. High-speed videos are used to assess the visual collapsing cavities. And it is complemented by paint test or erosive material test. But these methods are expensive. With the development of computational fluid dynamics (CFD), numerical methods become an attractive alternative. Ochiai et al. [31] used a Lagrangian method to assess the risk of cavitation erosion based on acoustic pressure emitted from bubbles. Peter et al. [32, 33] proposed a new method considering the microjet mechanism and applied the method to predict cavitation erosion around a hydrofoil. Pereira et al. [25, 34–36] used a macroscopic cavitation theory to assess the cavitation erosion risk in experiment and simulation. Usta et al. [37] compared the applicability of intensity function method, Gary level method, and erosive power method and predicted the erosible areas on a ship propeller.

The cavitation mechanism in the water-jet pumps was analyzed based on the RANS method and the DCM-SST turbulence model, and the cavitation erosion was predicted based on the macroscopic cavitation theory in this paper.

## 2. Experimental Setup

### 2.1. Pump Parameters

The main parameters of the water-jet pump are shown in Table 1.

### 2.2. Experimental Method [38]

The cavitation in the water-jet propulsion pump will destroy the energy exchange between the impeller and the liquid, which results in the decline of the external characteristics. The experiment in this paper was completed on the closed test bench of the water-jet pump of the 708th Research Institute of China State Shipbuilding Corporation by Long et al. [38]. NPSH*_a_* is gradually reduced until the pump head drops by 3% through reducing the pressure at the pump's inlet. High-speed photography technology was used to observe the cavitation structure during cavitation evolution through the plexiglass window in the impeller shell.

### 2.3. High-Speed Photography Acquisition System [38]

**Figure 1:**
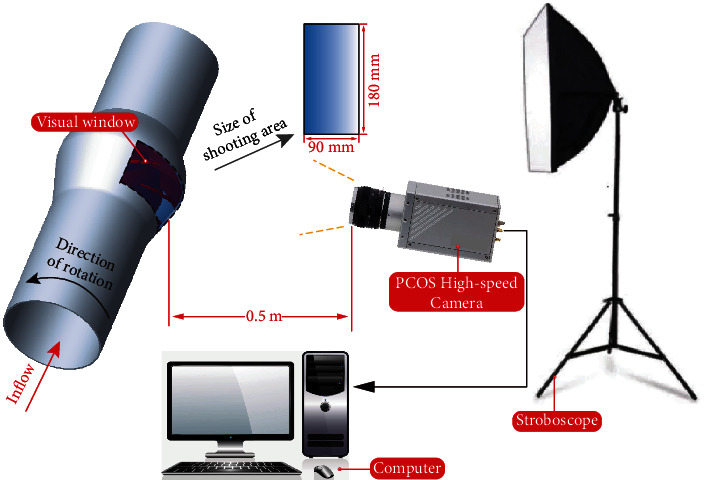
High-speed photography visualization system.

The high-speed photography visualization system is shown in Figure 1. The middle section of the impeller is perpendicular to the camera's longitudinal axis. The distance from the camera lens to the Plexiglas is about 0.5 m, and the size of the shooting area is about 90 mm × 180 mm. The high-speed camera PCOS achieves fast frame rates at high photosensitivity and high dynamic range, reaching 4467 fps with a full resolution of 1008 × 1008 pixels which can guarantee good image quality.

To capture the cavitation flow of the pump, the shooting frequency is determined as follows. If the rotating speed of the pump is *n* and the impeller is required to acquire an image once it rotates a certain degree *α*, the shooting frequency *f* is

(1) $$\begin{array}{c} f=\frac{n\times 360/60}{\alpha }, \end{array}$$

where *n* is the impeller rotating speed (r/min) and *α* is the impeller rotation angle (°).

In this paper, the camera is set as shooting one image for each 2° of the impeller rotation.

## 3. Numerical Simulation Methods

### 3.1. Continuity Equation and Momentum Equation

In the simulation, the vapor-liquid two-phase flow is generally assumed to be homogeneous. The Navier-Stokes equation based on the Newtonian fluid is used in this simulation [39, 40]. The equation is formulated in the Cartesian coordinate system as [23]

(2) $$\begin{array}{c} \frac{\partial \rho_{m}}{\partial t}+\frac{\partial \left(\rho_{m}u_{j}\right)}{\partial x_{j}}=0,\\ \frac{\partial \left(\rho_{m}u_{i}\right)}{\partial t}+\frac{\partial \left(\rho_{m}u_{i}u_{j}\right)}{\partial x_{j}}=-\frac{\partial p}{\partial x_{i}}+\frac{\partial }{\partial x_{j}}\times \left[\left(\mu_{m}+\mu_{T}\right)\left(\frac{\partial u_{i}}{\partial x_{j}}+\frac{\partial u_{j}}{\partial x_{i}}-\frac{2}{3}\frac{\partial u_{i}}{\partial xj}\delta_{ij}\right)\right],\\ \frac{\partial \rho_{l}\alpha_{l}}{\partial t}+\frac{\partial \left(\rho_{l}\alpha_{l}u_{j}\right)}{\partial x_{j}}=m^{+}+m^{-},\\ \rho_{m}=\rho_{l}\alpha_{l}+\rho_{v}\alpha_{v},\\ \mu_{m}=\mu_{l}\alpha_{l}+\mu_{v}\alpha_{v}, \end{array}$$

where the subscript *i*, *j* indicates the coordinate direction, *u* indicates the velocity, *p* indicates the pressure, *ρ*_*l*_ indicates the liquid density, *ρ*_*v*_ indicates the vapor density, *α*_*v*_ indicates the vapor volume fraction, *α*_*l*_ indicates the liquid volume fraction, *μ*_*l*_ indicates the liquid laminar viscosity, *μ*_*v*_ indicates the vapor laminar viscosity, *μ*_*T*_ indicates the turbulent viscosity, *m*^+^ indicates the vapor condensation rate, and *m*^−^ indicates the vapor evaporation rate. *ρ*_*m*_ indicates vapor-liquid mixed-phase density, and *μ*_*m*_ indicates vapor-liquid mixed-phase laminar viscosity.

### 3.2. Cavitation Model

The ZGB model [41, 42] is a cavitation model based on the mass transport equation, which describes the cavitation phase change process mainly by establishing the transport relationship between the vapor and liquid phases. Its evaporation rate and condensation rate are defined as follows:

(3) $$\begin{array}{c} m^{+}=C_{\text{dest}}\frac{3\alpha_{\text{nuc}}\left(1-\alpha_{v}\right)\rho_{v}}{R_{\text{B}}}{\left(\frac{2}{3}\frac{p_{v}-p}{\rho_{l}}\right)}^{1/2},\;p<p_{v},\\ m^{-}=-C_{\text{prod}}\frac{3\alpha_{v}\rho_{v}}{R_{\text{B}}}{\left(\frac{2}{3}\frac{p-p_{v}}{\rho_{l}}\right)}^{1/2},\;p>p_{v}, \end{array}$$

where *R*_B_ is the bubble radius, *α*_nuc_ is the volume fraction of gas nuclei, *p*_*v*_ is the saturated vapor pressure, *C*_prod_ is the rates of steam condensation when the local static pressure is greater than the saturation vapor pressure, and *C*_dest_ is the rate of steam evaporation when the local static pressure is lower than the saturation vapor pressure. The values of the coefficients in the model are [43] *R*_*B*_ = 1 × 10^−6^*m*, *C*_prod_ = 0.01, *C*_dest_ = 50, and *α*_nuc_ = 1 × 10^−4^.

### 3.3. Turbulence Model

This simulation is based on the *SSTk* − *ω*turbulence model [50]. To more accurately simulate the development of cavitation in the centrifugal pump, the compressibility of the mixing of the vapor and liquid phases is considered, and the mixing density is corrected using the *DCM* method. Turbulent viscosity is defined as follows:

(4) $$\begin{array}{c} f_{\text{DCM}}=\rho_{v}+{\left(\frac{\rho_{m}-\rho_{v}}{\rho_{l}-\rho_{v}}\right)}^{N}\cdot \left(\rho_{l}-\rho_{v}\right),\\ \mu_{\text{T}‐\text{DCM}}=\frac{C_{\mu }k^{2}}{\varepsilon }f_{\text{DCM}}, \end{array}$$

where *N* is taken as 10 according to literature recommendations [43, 44].

### 3.4. Boundary Conditions and Grid Setup

**Figure 2:**
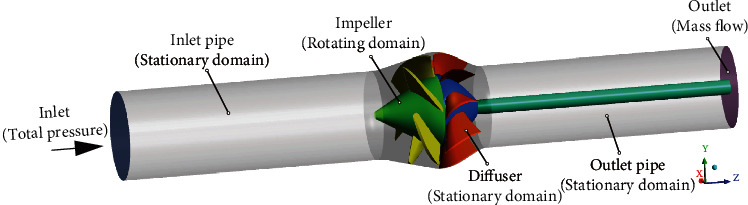
Calculation domain and boundary conditions.

The calculation parameters are shown in Table 2.  Figure 2 shows the calculation domain and boundary conditions. The inlet of the calculation domain was set as the pressure inlet, and the outlet condition was mass flow rate. The domain of impeller was rotating with a speed of 1450 rpm, and other domains were static domains. Inlet and outlet extension pipes were added to avoid the large difference between experiment and simulation caused by backflow. The wall condition was set as *nonslip wall*. The residual was set to be 1*e* − 04. Total calculation time was set as 0.12 s, and timestep was set to be 2.73*e* − 05 s.

**Figure 3:**
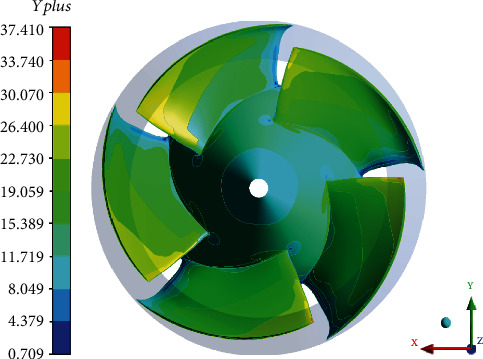
Distribution of *Yplus.*

**Figure 4:**
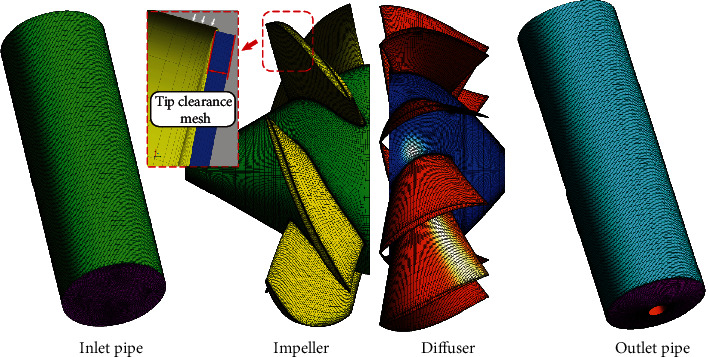
The detail of grid.

After completing the 3D modeling, the inlet pipe and the outlet pipe were divided into structural grids by software *ICEM*. Impeller and diffuser grids were structured using software *TurboGrid*. The leading and trailing edges of the blades are elliptical. The spline curve is used to fit the contour of each layer of the grids. The tip clearance of the grid was set to 0.3 mm. The grid of tip clearance region was divided into 10 layers to obtain a better flow field. Regarding the grid-independent inspection, the water-jet propulsion pump studied in this paper is similar to the calculation model of Huang et al. [20–23] in terms of geometrical dimensions and operating conditions. In Huang's research, the mesh-independence check was made for the impeller, and it was pointed out that the mesh of the impeller is larger than 1.06 million to ensure the calculation accuracy. Considering the requirements of the turbulence model for the boundary layer, the *y*+ of near-wall surface shown in Figure 3 was adjusted below 40. The number of grids used in this paper is shown in Table 3, and the details of the grids are shown in Figure 4.

## 4. Analysis of Results

### 4.1. Cavitation Performance and Analysis

**Figure 5:**
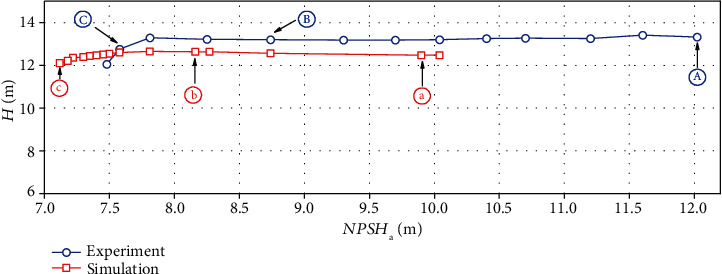
Comparison between simulated and experimental cavitation performance curves.

**Figure 6:**
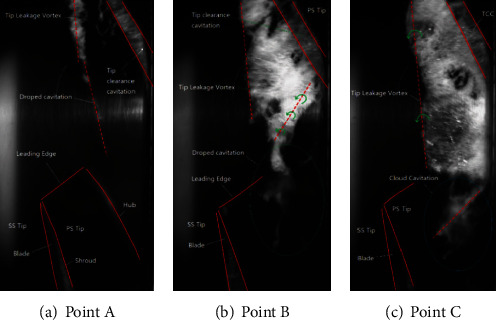
The cavity flow structures at different cavitation conditions [15].

The net positive suction head and corresponding head under the design flow rate are obtained. The cavitation characteristic curve was obtained by reducing the inlet pressure gradually. The experimental and simulation results are shown in Figure 5. This water-jet propulsion pump's rated head is 13 m, and the simulated result is 12.5 m. The relative error is 3.8%, and this error can prove that the simulation is reliable. When the simulated head drops by 3%, NPSH*_a_* is 7.15 m, while NPSH*_a_* is 7.58 m in the experiment for the same situation. This difference may be because noncondensable gases, thermodynamic effects, and other factors were ignored. The existence of this difference does not affect capturing the flow characteristics in the pump by simulation. The cavitation image captured in the experiment is shown in Figure 6. Figures 5 and 6 are compared and analyzed. As can be seen in Figure 6(a), in the initial stage of cavitation, the head of point A basically does not change. It is generally believed that there is little cavitation in the blade channel at this operating condition, but the cavitation in the tip clearance can be seen from the image. As the inlet pressure decreases, the head curve begins to change. During the evolution of cavitation, point B on the cavitation performance curve was selected, as the initial cavitation. As the net positive suction head decreases, the tip leakage vortex (TLV) area becomes larger, and at the same time, the cavity grows on the suction surface, as shown in Figure 6(b). When the head drops by 3%, the point C on the cavitation performance curve is called the critical cavitation point. a~c on the simulated cavitation performance curve were selected to mark the cavitation situations corresponding to A~C on the experimental cavitation curve. The corresponding NPSH*_a_* of point A is 9.90 m, point b is 8.16 m, and point c is 7.15 m.

**Figure 7:**
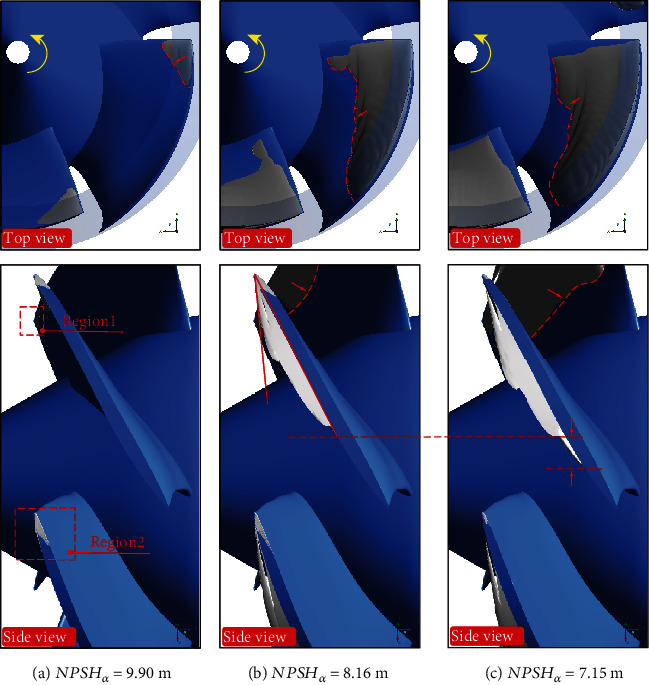
Simulated cavitation structures at different NPSH*_a_*.

**Figure 8:**
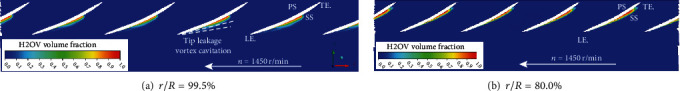
Contours of vapor volume fraction at NPSH_*a*_ = 7.15 m.

**Figure 9:**
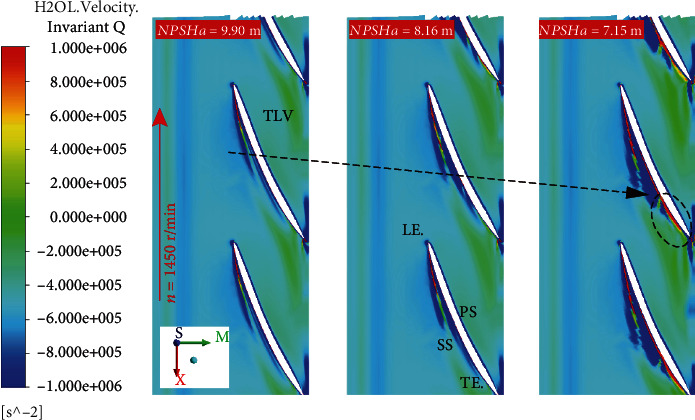
Distribution of vortex core by *Qinvariant* method (*r*/*R* = 99.5%).

**Figure 10:**
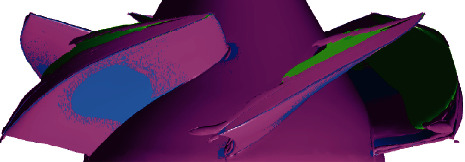
Cavitation vortex structure at NPSH_*a*_ = 7.15 m. (The vortex center region is shown as pink isosurface of *Q* = 5*e* + 05 s^−2^, and the green areas represent cavitation structures.)

**Figure 11:**
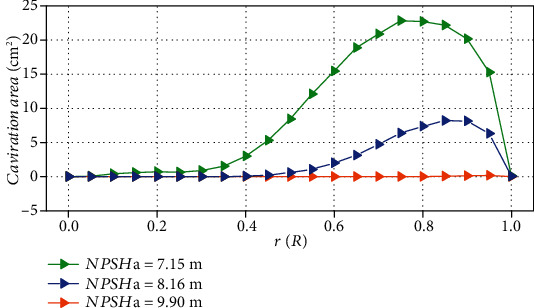
Cavity area at different NPSH_*a*_.

Figure 7 shows the cavitation structures under different net positive suction heads captured by the simulation, which were represented by gray isosurface with vapor volume fraction of 10%. Observed from the side view, the simulated cavity structure is basically consistent with the experimental results. When NPSH*_a_* is 9.90 m, tiny cavitation appears near the tip on the suction surface of the blades, as shown in region 1 of the figure. At the same time, the cavitation in the tip clearance was also captured, as shown in region 2 of the figure. At this situation, the appearance of cavitation has no effect on performance. As the net positive suction head drops to 8.16 m, the cavity develops from the tip to the root of the blades. In the flow direction, it covers most of the blade surface. It can be observed that there is “gap” in the cavitation isosurface near the leading edge of blades. As the net positive suction head continues to drop, it can be observed that the “gap” is more obvious, and the cavitation appears to be stratified. Compared with Figure 6, it can be considered that this delamination may be caused by the tip leakage vortex (TLV) (this will be analyzed in Figures 8–10). The existence of leakage flow causes tip leakage vortex cavitation (TLVC). The local pressure near the blades is lower than the saturated vapor pressure, and cavitation appears on the suction surface. The different cavitation structures gathered together, resulting in a decrease in pump performance.  Figure 11 shows the change of the cavitation area at different circumferential positions in the blade channel under design conditions. It can be seen from the figure that as *r*/*R* increases, the cavitation area first increases and then decreases. When NPSH_*a*_ = 9.90 m, the maximum cavitation area of 0.186 cm^2^ appears at *r*/*R* = 0.95; when NPSH_*a*_ = 8.16 m, the maximum value of 8.20 cm^2^ appears at *r*/*R* = 0.85; when NPSH_a_ = 7.15 m, the maximum cavitation area of 22.8 cm^2^ appears at *r*/*R* = 0.75. This phenomenon verifies that the cavitation in the water-jet propulsion pump mainly occurs in the area near the tip of the blades. The cavitation coverage area expands from the tip to the root as the net positive suction head drops. The maximum cavitation area in the impeller domain moves from the tip to the root of the blades.

The evolution of cavitation is closely related to the flow field structure. There are many ways to identify the vortex structure [45]. To better analyze the structure of the flow field, the *Q*-criterion is introduced to describe the vortex:

(5) $$\begin{array}{c} Q=\frac{1}{2}\left({\left\|\Omega \right\|}^{2}-{\left\|\text{S}\right\|}^{2}\right)=\frac{1}{2}\left(\Omega_{ij}\Omega_{ji}-S_{ij}S_{ji}\right), \end{array}$$

where *Q* represents the second-order invariant of the velocity gradient tensor under Galileo transformation. *Ω* represents the vorticity tensor and *S* represents the deformation rate tensor, which are, respectively, defined as

(6) $$\begin{array}{c} S_{ij}=\frac{1}{2}\left(\frac{\partial u_{i}}{\partial x_{j}}+\frac{\partial u_{j}}{\partial x_{i}}\right),\\ \Omega_{ij}=\frac{1}{2}\left(\frac{\partial u_{i}}{\partial x_{j}}-\frac{\partial u_{j}}{\partial x_{i}}\right), \end{array}$$

where *i* and *j*, respectively, represent different directions. It can be seen from the equation that *Q* is equal to the value of vorticity tensor subtracting the deformation rate tensor. When *Q* > 0, it means that the tendency of rotation is greater than the tendency of axial deformation. At this time, it can be considered that the flow in this area is dominated by eddy flow.

**Figure 12:**
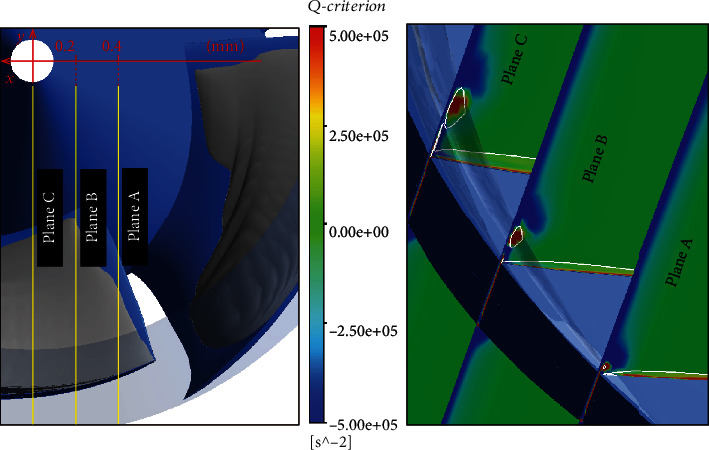
The relationship between cavitation and vortex structure. (The white line represents the boundary of vapor-liquid phase.)

Figure 8 shows the cavity distribution on different *r*/*R* spanwise sections. When *r*/*R* = 99.5%, the cavity in the impeller is divided into two parts: the attached cavity on the suction surface of the blades and the elongated vortex cavitation appearing at the leading edge of blades. When *r*/*R* = 80%, only attached cavity exists on the blades. When *r*/*R* is 80%, the cavity length is greater than that when *r*/*R* is 99.5%.  Figure 9 shows the distribution of *Q* in the spanwise section at different *r*/*R* locations. In the far-field region, the *Q* values are negative. The results show that the trend of axial deformation dominates the distribution of the *Q* value, due to the rotation of the impeller. Also, it can be seen from the figure that the method can accurately identify the core of the tip leakage vortex (TLV) with a higher positive value on the blades' suction side (SS). The TLV region gradually becomes longer with the decreases of NPSH*_a_*. The *Q* value near the initial position of the TLV is the highest. As the TLV moves away from the leading edge, the *Q* value gradually decreases. But its value is still positive, and the flow is still dominated by the rotational effect. There is an area with extremely low value of *Q* around the TLV structure, which reflects the restriction of the rotation effect and the deformation effect. At the position corresponding to the TLV structure in Figure 8(a), there is a slender cavitation on the SS of the blades, and the shapes are basically similar. This cavitation is caused by tip leakage. As shown in Figure 10, the TLV structure seems a “boundary” that defines the cavitation on the leading edge of the blades. The cavitation only develops in this boundary. This is consistent with the results observed through experimental results in Figure 6. Three planes were set to analyze the relationship between tip leakage vortex and cavity structure as shown in Figure 12. The area of tip leakage vortex is small on plane A. The cavitation structure is located at the vortex core. And the value of *Q* beyond the phase boundary is also high. The development of tip leakage vortex will be affected by the rotation of the impeller. From plane A to plane C, the TLV region gradually became bigger and the max value of *Q* reduced. The position of the phase boundary moves away from the vortex core area.

### 4.2. Prediction of Cavitation Erosion

**Figure 13:**
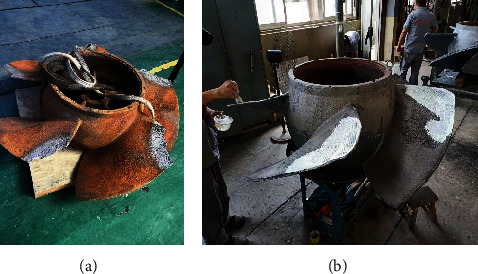
Distribution of cavitation erosion on pumps.

Cavitation will cause damage to the pump blades, and the erosion profile in practical engineering is shown in Figure 13. Cavitation erosion is a microscopic and transient process, but it is also affected by macroscopic flow conditions. From the energy point of view, the collapse of the cavitation bubble will produce a pressure wave. This pressure wave is one of the factors that cause cavitation damage. Based on the hypothesis of Pereira et al. [25, 34], the potential energy of the cavity structure is defined as

(7) $$\begin{array}{c} E_{\text{pot}}=\Delta p\times V_{\text{vap}},\\ \Delta p=\left(p-p_{\text{sat}}\right). \end{array}$$

The cavitation volume is reduced when cavity collapses, and the pressure wave is released. The erosive power function *p*_pot_ can be defined as [25, 27–30, 33, 35, 36, 40, 46]

(8) $$\begin{array}{c} p_{\text{pot}}=\frac{\partial E_{\text{pot}}}{\partial t}=\Delta p\times \frac{\partial V_{\text{vap}}}{\partial t}+V_{\text{vap}}\times \frac{\partial \Delta p}{\partial t}=\left(p-p_{\text{sat}}\right)\times V_{\text{cell}}\times \frac{\partial \alpha }{\partial t}+V_{\text{cell}}\times \alpha \times \frac{\partial p}{\partial t},\;\text{if }p_{\text{pot}}\geq \varepsilon , \end{array}$$

(9) $$\begin{array}{c} \alpha =\frac{V_{\text{vap}}}{V_{\text{cell}}}=\frac{\rho -\rho_{l}}{\rho_{v}-\rho_{l}}, \end{array}$$

where *p* is the pressure surrounding the vapor (Pa), *p*_sat_ is the saturated vapor pressure (Pa), *V*_vap_ is the vapor volume (m^3^), *V*_cell_ is the volume of the grid (m^3^), *α* is the vapor volume fraction, and *ε* is the threshold.

**Figure 14:**
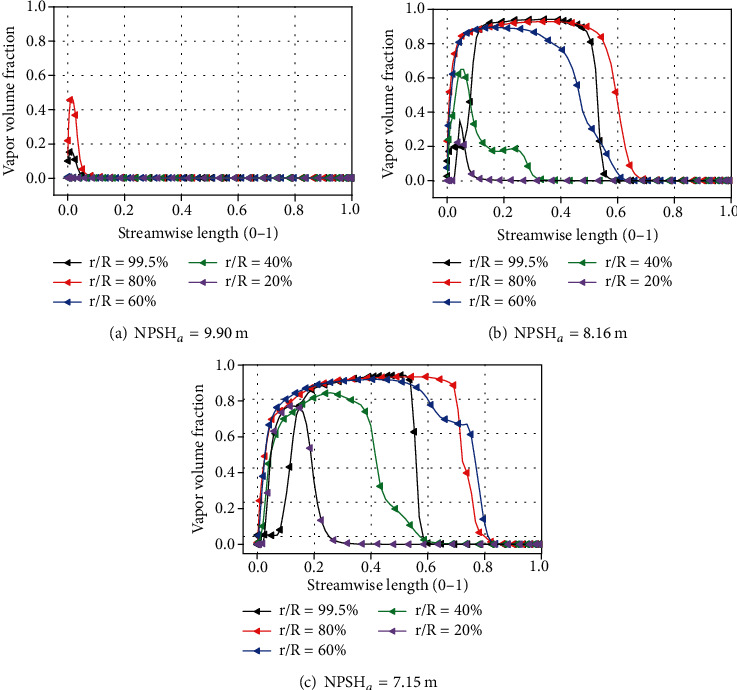
Vapor volume fraction at different spans.

**Figure 15:**
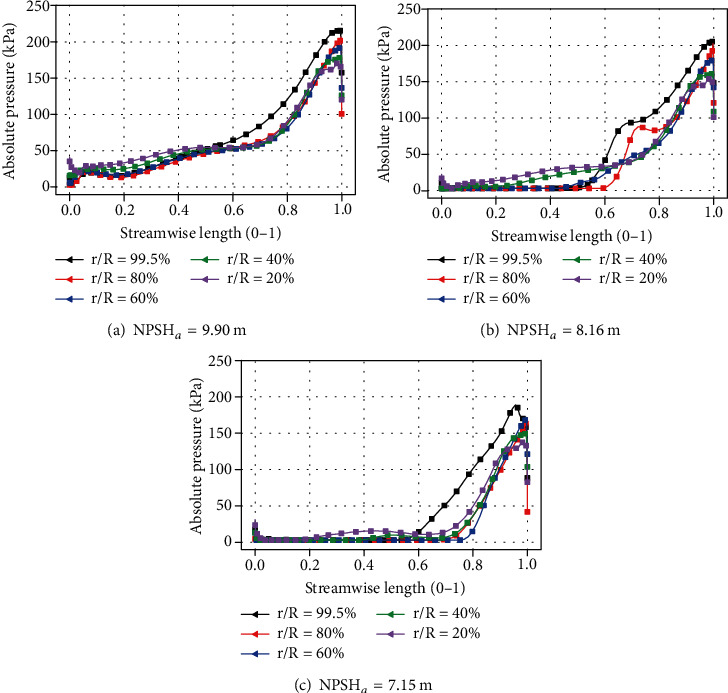
Absolute pressure at different spans.

**Figure 16:**
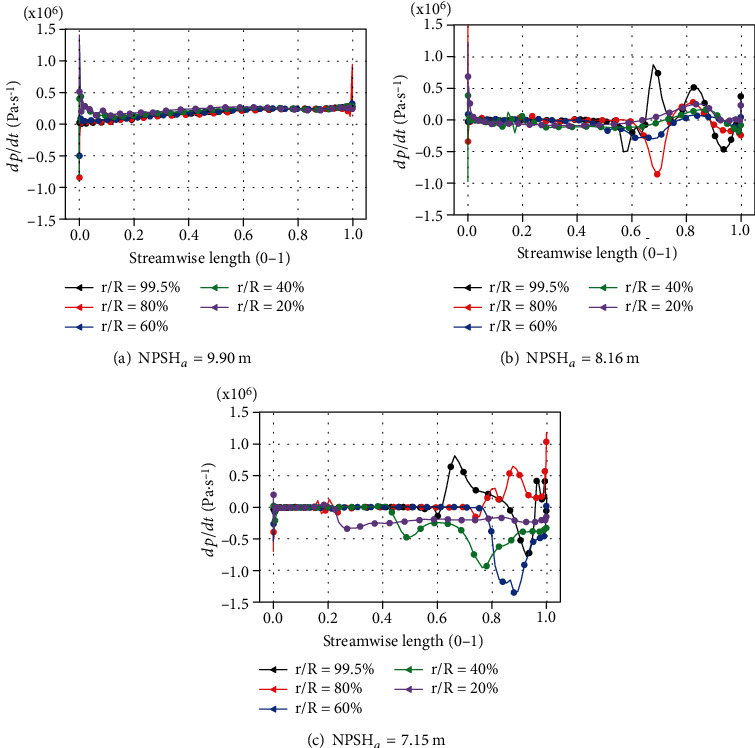
*dp*/*dt* at different spans.

There are 5 key parameters in equation (8), which are *p*, *α*, *∂p*/*∂t*, and *∂α*/*∂t*. In order to better analyze the distribution of related parameters and their influence on cavitation erosion prediction, the distribution information of related parameters at different spans was extracted and drawn in Figures 14–16.  Figure 14 shows the cavitation volume fraction distribution under rated flow rate. When NPSH_*a*_ = 9.90 m, there is a tiny cavity near the tip at *r*/*R* = 80% and 99.5%. At the position of *r*/*R* = 80%, the cavity coverage length is longer than that at *r*/*R* = 99.5%. But near the root at *r*/*R* = 20%, 40%, and 60%, no cavity exists. When the net positive suction head decreases to 8.16 m, the cavity volume at *r*/*R* = 80% and 99.5% near the tip increased rapidly. In the flow direction, the length of the cavity coverage becomes longer, but the length of the cavity at the position of *r*/*R* = 80% is still greater than that at *r*/*R* = 99.5%. Cavitation also exists at *r*/*R* = 20%, 40%, and 60%. With a change of *r*/*R* from tip to the root, both the vapor content and the coverage length decreased. When NPSH_*a*_ = 7.15 m, the coverage length of cavity at different *r*/*R* positions becomes longer. The position with the longest cavity is at *r*/*R* = 60%. The maximum vapor content at the positions of *r*/*R* = 20%, 40%, and 60% all becomes larger. Comparing the vapor volume fraction distribution under different NPSH*_a_*, it can also be observed that the leading edge of the blade at *r*/*R* = 99.5% has lower vapor content. The cavitation volumes at the leading edge (LE) of the blade at *r*/*R* = 80% position rise rapidly along the flow direction. The cause of this change may be due to the existence of the tip leakage vortex.

The pressure distribution on the blade surface will directly affect the performance of blades [51].  Figure 15 shows the absolute pressure distribution on the suction surface of the blade under rated flow rate. The pressure gradually increases along with the flow direction. The absolute pressure near the root position when streamwise length ranges from 0 to 0.5 is greater than that near the tip position. But when streamwise length ranges from 0.5 to 1.0, the result is opposite. As the inlet pressure drops, the maximum pressure value on the blade gradually decreases. Due to the coverage of the cavity, the area where the absolute pressure is less than 50 kPa gradually becomes larger.

*dp*/*dt* can be used as an indicator for cavitation prediction [47].  Figure 16 shows the distribution of *dp*/*dt* under the rated flow rate. When NPSH_*a*_ = 9.90 m, the variation of *dp*/*dt* mainly appears at the leading edge and trailing edge of the blades. The maximum value of ∣*dp*/*dt*∣ appearing on the leading edge is greater than that appearing on the tailing edge. As the net positive suction head decreases to 8.16 m, the maximum value of ∣*dp*/*dt*∣ still appears at the leading edge of the blade. But the fluctuation area of *dp*/*dt* near the trailing edge of the blade begins to become larger. When NPSH*_a_* decreases to 7.15 m, the area with large fluctuations of *dp*/*dt* on the suction surface of the blade expanded, and the maximum value of ∣*dp*/*dt*∣ on the blade also increased from 0.87*e* + 06 to 1.34*e* + 06. This phenomenon shows that as the net positive suction head drops, the pressure fluctuations gradually become larger.

**Figure 17:**
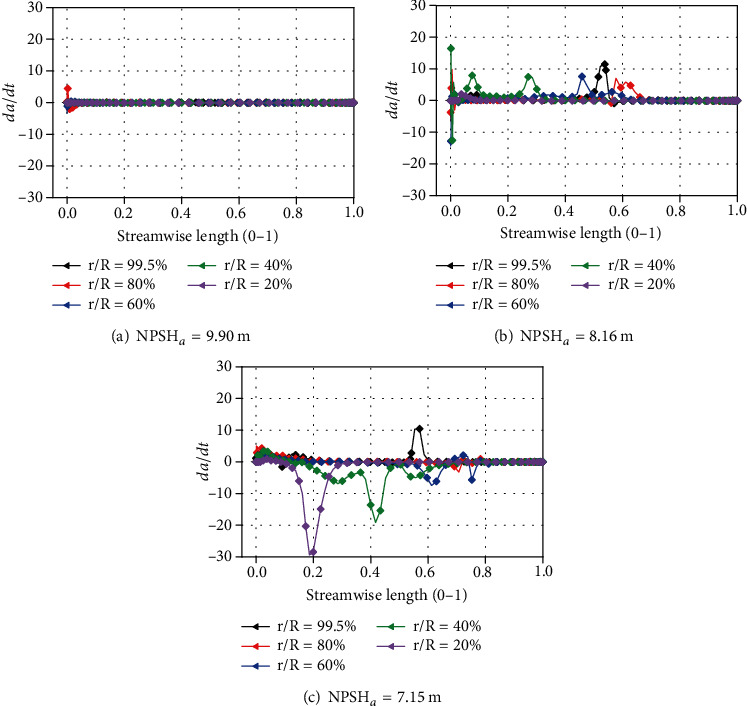
*dα*/*dt* at different spans.

Figure 17 shows the distribution of *dα*/*dt* under the rated flow rate. The distribution of *dα*/*dt* is related to the location of the cavity. When NPSH_*a*_ = 9.90 m, the sudden change of *dα*/*dt* mainly appears on the leading edge of the blade, and when NPSH_*a*_ = 8.16 m, the sudden change of *dα*/*dt* appears when streamwise length ranges from 0 to 0.7. The sudden change of *dα*/*dt* appears when streamwise length ranges from 0 to 0.8 if NPSH*_a_* is 7.15 m. This distribution is consistent with the results in Figure 7. It can also be seen that as the net positive suction head decreases, the maximum value of ∣*dα*/*dt*∣ also increases from 11.5 to 29.6. This phenomenon shows that as the net positive suction head decreases, the fluctuation of the vapor content gradually increases.

The unsteady simulation captured the transient contours of vapor volume fraction, *dp*/*dt*, *dα*/*dt*, and *p*_pot_. The erosion caused by cavitation is the result of a long-duration accumulation. To better predict the erodible region, the *Matlab* code was used to process the images into time-averaged results. For ease of description, the abbreviated form is expressed as follows: TAV (time-averaged vapor volume fraction), TAA (time-averaged *dα*/*dt*), TAP (time-averaged *dp*/*dt*), and TAPP (time-averaged *p*_pot_).

72 snapshots were set during one rotation. The image processing method refers to the singular value decomposition (SVD) [48]. An image can be interpreted as a matrix by linear transformation, described by eigenvalues and eigenvectors. If the flow variables *U*(*t*, *x*) (pressure, velocity, etc.) during a period of time *t* are known (where *x* is the space vector of the flow field), the time information of *M* moments is stored in the form of row vectors and the spatial information of *N* nodes is stored in the form of column vectors. The matrix can be expressed as

(10) $$\begin{array}{c} U\left(t_{i},x_{j}\right)=\left[\begin{array}{ccccc} u\left(t_{1},x_{1}\right) & u\left(t_{1},x_{2}\right) & u\left(t_{1},x_{3}\right) & .. & u\left(t_{1},x_{N}\right)\\ u\left(t_{2},x_{1}\right) & u\left(t_{2},x_{2}\right) & u\left(t_{2},x_{3}\right) & .. & u\left(t_{2},x_{N}\right)\\ . & . & . & . & .\\ . & . & . & . & .\\ u\left(t_{M},x_{1}\right) & u\left(t_{M},x_{2}\right) & u\left(t_{M},x_{3}\right) & .. & u\left(t_{M},x_{N}\right) \end{array}\right]. \end{array}$$

The time average of the transient variables at each node can be expressed as

(11) $$\begin{array}{c} \bar{U}\left(x_{J}\right)=\frac{1}{M}\overset{M}{\underset{i=1}{\sum }}U\left(t_{i},x_{j}\right). \end{array}$$

**Figure 18:**
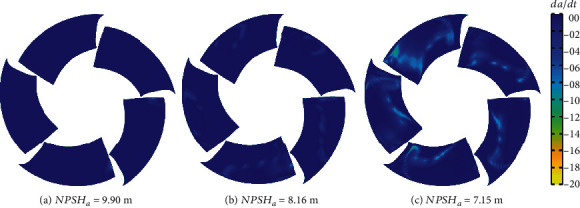
Time-averaged *dα*/*dt*.

Figure 18 shows the TAA distribution after one rotation at rated flow rate. Based on the hypothesis of macroscopic cavitation, pressure waves will be generated when the vapor content decreases. The threshold of the *dα*/*dt* parameter was set to (−20, 0). When NPSH*_a_* is 9.90 m, the large value of ∣TAA∣ appears near the leading edge of the blade near the root. Compared with the other two NPSH*_a_*, the maximum value of ∣TAA∣ is smaller and the distribution area is not obvious. As the net positive suction head decreases, the TAA distribution area gradually becomes larger, expanding from the leading edge to the tailing edge along the flow direction and from the tip to the root along the span direction. The maximum value of ∣TAA∣ on the suction surface of the blade gradually becomes larger. Compared to Figure 17, it can be seen that the distribution of TAA matches with the shape of the cavity coverage line.

**Figure 19:**
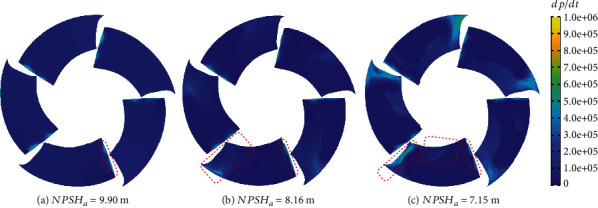
Time-averaged *dp*/*dt*.

A pressure pulse with positive amplitude is an important indicator of cavitation erosion [47]. The threshold of the *dp*/*dt* parameter was set to (0, 1.0*e* + 06).  Figure 19 shows the TAP distribution after one rotation at rated flow rate. It can be seen from the figure that there is a higher value on the leading edge of the blade after the time-averaged treatment. The occurrence of this area is related to the impact of the incoming flow on the blades. At the rear part of the blade, the distribution of TAP is related to the distribution of cavities. The high TAP in the midstream without cavitation is concentrated and is also close to the root of the blade, as shown in Figure 19(c). As the net positive suction head decreases, the maximum value of TAP gradually increases, and it gradually moves to the trailing edge.

**Figure 20:**
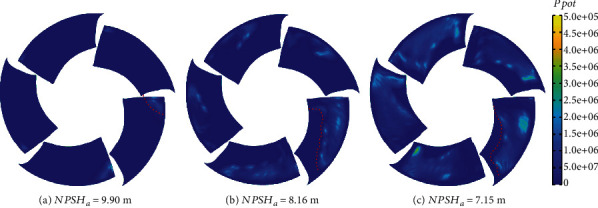
Time-averaged *p*_pot_.

**Figure 21:**
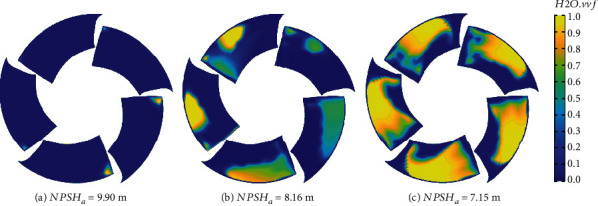
Time-averaged vapor volume fraction.

Based on the distribution of TAA and TAP, the time-averaged distribution of erosion power after one rotation is obtained based on equation (8), as shown in Figure 20. The shape of the erosion region in the figure matches with the cavitation coverage area in Figure 21. This is consistent with the conclusions obtained by other scholars [49]. The result of cavitation prediction is also consistent with the phenomenon in Figure 13. The light-colored areas on the blades represent cavitation erosion in the figure. Cavitation erosion first appeared near the tip of the blade and developed to the trailing edge of the blade along the flow direction. There is more cavitation erosion near the tip of the blades. And cavitation erosion basically does not exist on the blade roots. The simulation results showed the same trend. When NPSH*_a_* is 9.90 m, the region of cavitation erosion appears at the leading edge near the tip with a near-triangular shape. The erosion area expanded when NPSH*_a_* is 8.16 m and mainly distributed near the tip. The high value distribution of TAPP is basically located near the cavity closure. As the net positive suction head continues to decrease, the erosion area expands toward the trailing edge of the blade along the flow direction and expands toward the root of the blade along the span direction. It can be observed from Figure 20 that as NPSH*_a_* decreases, the maximum value of TAPP gradually increases, and the risk of erosion becomes more serious.

## 5. Conclusion

Simulated and experimental methods were used to study the unsteady flow mechanism and cavitation erosion in a water-jet pump, and the following conclusions are obtained:

1. The head of the water-jet pump calculated by using the DCM-SST turbulence model is 12.48 m. The calculation error at the rated head is 3.8%
2. With the decrease of the net positive suction head, the position where the severe cavitation appears in the impeller domain gradually moves from the tip to the root
3. The erosion region obtained by the erosion power method is similar to the cavity profile. As the net positive suction head decreases, the erodible area and the erosion intensity become larger

## Figures and Tables

**Table 1:** Design parameters of the test pump.

Design parameter	Design value
Flow rate *Q* (m^3^/s)	0.46
Head *H* (m)	13
Rotating speed *n* (r/min)	1450
Power *N* (kW)	70
Specific speed *n*_s_	524.3
Impeller inlet diameter *D*_j_ (mm)	270

**Table 2:** Calculation parameters.

Parameter	Value
Temperature (°C)	25
Saturated vapor pressure (Pa)	3169.93
Density of water (kg/m^3^)	997.003
Dynamic viscosity of liquid phase (Pa·s)	8.9*e* − 04
Density of vapor (kg/m^3^)	2.3*e* − 02
Dynamic viscosity of vapor phase (Pa·s)	9.87*e* − 06

**Table 3:** Mesh number of calculation domain.

Calculation domain	Mesh number
Impeller	1729170
Diffuser	1579710
Inlet pipe	626640
Outlet pipe	590236
Total mesh number	4525756

## Data Availability

The data that support the findings of this study are available from the corresponding author upon reasonable request.

## References

[1] Park W., Jang J. H., Chun H. H., Kim M. C. (2005). Numerical flow and performance analysis of waterjet propulsion system. *Ocean Engineering*.

[2] Wang Y., Hao E., Zhao X., Xue Y., An Y., Zhou H. (2022). Effect of microstructure evolution of Ti6Al4V alloy on its cavitation erosion and corrosion resistance in artificial seawater. *Journal of Materials Science & Technology*.

[3] Chen Z., Hu H., Guo X., Zheng Y. (2022). Effect of groove depth on the slurry erosion of V-shaped grooved surfaces. *Wear*.

[4] Zhang L. M., Li Z. X., Hu J. X. (2021). Understanding the roles of deformation-induced martensite of 304 stainless steel in different stages of cavitation erosion. *Tribology International*.

[5] Song Q. N., Tong Y., Li H. L. (2021). Corrosion and cavitation erosion resistance enhancement of cast Ni-Al bronze by laser surface melting. *Journal of Iron and Steel Research International*.

[6] Hong S., Lin J., Wu Y. (2021). Cavitation erosion characteristics at various flow velocities in NaCl medium of carbide-based cermet coatings prepared by HVOF spraying. *Ceramics International*.

[7] Hong S., Wu Y., Wu J. (2021). Microstructure and cavitation erosion behavior of HVOF sprayed ceramic-metal composite coatings for application in hydro-turbines. *Renewable Energy*.

[8] Wang Y., Liu H., Yuan S., Tan M., Wang K. (2012). Experimental testing on cavitation vibration and noise of centrifugal pumps under off-design conditions. *Transactions of the Chinese Society of Agricultural Engineering*.

[9] Wang H., Long B., Wang C., Han C., Li L. (2020). Effects of the impeller blade with a slot structure on the centrifugal pump performance. *Energies*.

[10] Brennen C. E. (2013). *Cavitation and Bubble Dynamic*.

[11] Zhou W., Qiu N., Wang L., Gao B., Liu D. (2018). Dynamic analysis of a planar multi-stage centrifugal pump rotor system based on a novel coupled model. *Journal of Sound and Vibration*.

[12] Tan D., Li Y., Wilkes I., Vagnoni E., Miorini R. L., Katz J. (2015). Experimental investigation of the role of large scale Cavitating Vortical Structures in performance breakdown of an axial Waterjet pump. *Journal of Fluids Engineering*.

[13] Motley M., Savander B., Young Y. (2014). Influence of Spatially Varying Flow on the Dynamic Response of a Waterjet inside an SES. *International Journal of Rotating Machinery*.

[14] Yun L., Yan Z., Jianping C., Rongsheng Z., Dezhong W. (2021). A cavitation performance prediction method for pumps: Part2-sensitivity and accuracy. *Nuclear Engineering and Technology*.

[15] Long Y., Feng C., Wang L., Wang D., Cai Y., Zhu R. (2019). Experiment on cavitation flow in critical cavitation condition of water-jet propulsion pump. *Journal of Beijing University of Aeronautics and Astronautics*.

[16] Wu H., Tan D., Miorini R. L., Katz J. (2011). Three-dimensional flow structures and associated turbulence in the tip region of a waterjet pump rotor blade. *Experiments in Fluids*.

[17] Lindau J. W., Boger D. A., Medvitz R. B., Kunz R. F. (2005). Propeller cavitation breakdown analysis. *Journal of Fluids Engineering*.

[18] Katz J. (1984). Cavitation phenomena within regions of flow separation. *Journal of Fluid Mechanics*.

[19] Guo Q., Huang X., Qiu B. (2019). Numerical investigation of the blade tip leakage vortex cavitation in a waterjet pump. *Ocean Engineering*.

[20] Huang R., Ji B., Luo X., Zhai Z., Zhou J. (2015). Numerical investigation of cavitation-vortex interaction in a mixed-flow water-jet pump. *Journal of Mechanical Science and Technology*.

[21] Huang R., Ye W., Dai Y. (2020). Investigations into the unsteady internal flow characteristics for a waterjet propulsion system at different cruising speeds. *Ocean Engineering*.

[22] Huang R., Yu A., Ji B., Zhou J., Zhai Z., Luo X. Cavitating flow features in a water-jet pump under different upstream conditions.

[23] Huang R., Dai Y., Luo X., Wang Y., Huang C. (2019). Multi-objective optimization of the flush-type intake duct for a waterjet propulsion system. *Ocean Engineering*.

[24] Xu S., Long X., Jin B., Li G. (2020). Investigation on the mechanism between vortex and cavitation in an axial water-jet pump. *Journal of Harbin Engineering University*.

[25] Qiu N., Zhou W., Che B., Wu D., Wang L., Zhu H. (2020). Effects of microvortex generators on cavitation erosion by changing periodic shedding into new structures. *Physics of Fluids*.

[26] Momma T., Lichtarowicz A. (1995). A study of pressures and erosion produced by collapsing cavitation. *Wear*.

[27] Franc J., Riondet M., Karimi A., Chahine G. L. (2011). Impact load measurements in an erosive cavitating flow. *Journal of Fluids Engineering*.

[28] Knapp R. (1955). Recent investigations of the mechanics of cavitation and cavitation damage. *Transactions of the ASME*.

[29] Dular M., Delgosha O. C., Petkovšek M. (2013). Observations of cavitation erosion pit formation. *Ultrasonics Sonochemistry*.

[30] Wang J., Liu H., Dular M. (2017). Experiment on cavitation erosion mechanism of centrifugal hydraulic cavitation generator. *Transactions of the Chinese Society of Agricultural Engineering*.

[31] Ochiai N., Iga Y., Nohmi M., Ikohagi T. (2013). Study of quantitative numerical prediction of cavitation erosion in cavitating flow. *Journal of Fluids Engineering*.

[32] Peters A., Sagar H., Lantermann U., el Moctar O. (2015). Numerical modelling and prediction of cavitation erosion. *Wear*.

[33] Peters A., Lantermann U., el Moctar O. (2018). Numerical prediction of cavitation erosion on a ship propeller in model- and full-scale. *Wear*.

[34] Pereira F. (1997). *Prediction de l'erosion de cavitation: approche energetique, [Ph.D. thesis]*.

[35] Fortes-Patella R., Challier G., Reboud J., Archer A. (2013). Energy balance in cavitation erosion: from bubble collapse to indentation of material surface. *Journal of Fluids Engineering*.

[36] Dular M., Coutier-Delgosha O. (2009). Numerical modelling of cavitation erosion. *International Journal for Numerical Methods in Fluids*.

[37] Usta O., Korkut E. (2019). Prediction of cavitation development and cavitation erosion on hydrofoils and propellers by detached eddy simulation. *Ocean Engineering*.

[38] Long Y., An C., Zhu R., Chen J. (2021). Research on hydrodynamics of high velocity regions in a water-jet pump based on experimental and numerical calculations at different cavitation conditions. *Physics of Fluids*.

[39] Wang H., Hu Q., Yang Y., Wang C. (2021). Performance differences of electrical submersible pump under variable Speed Schemes. *International Journal of Simulation Modelling*.

[40] Shi L., Zhu J., Tang F., Wang C. (2020). Multi-disciplinary optimization design of axial-flow pump impellers based on the approximation model. *Energies*.

[41] Zwart P., Gerber A., Belamri T. A Two-Phase Flow Model for Predicting Cavitation Dynamics.

[42] Zhou J., Zhao M., Wang C., Gao Z. (2021). Optimal design of diversion piers of lateral intake pumping station based on orthogonal test. *Shock and Vibration*.

[43] Chen L. (2020). *Study on Cloud Cavitation Control by Bionic Leading-Edge Tubercles on Hydrofoil*.

[44] Zhang L., Wang C., Zhang Y., Xiang W., He Z., Shi W. (2021). Numerical study of coupled flow in blocking pulsed jet impinging on a rotating wall. *Journal of the Brazilian Society of Mechanical Sciences and Engineering*.

[45] Zhao Y., Wang G. Y., Huang B., Hu C. L., Chen G. H., Wu Q. (2015). Vortex dynamic analysis of unsteady cavitating flows around a hydrofoil. *Journal of Ship Mechanics*.

[46] Zhu H., Qiu N., Wang C. (2021). Prediction of Cavitation Evolution and Cavitation Erosion on Centrifugal Pump Blades by the DCM-RNG Method. *Scanning*.

[47] Li Z., Pourquie M., van Terwisga T. (2014). Assessment of cavitation erosion with a URANS method. *Journal of Fluids Engineering*.

[48] Higham J. E., Brevis W., Keylock C. J. (2018). Implications of the selection of a particular modal decomposition technique for the analysis of shallow flows. *Journal of Hydraulic Research*.

[49] Wang J. (2015). *Numerical Simulation and Experimental Tests for Cavitation and Induced Erosion in Hydraulic Apparatus*.

[50] Che B. (2019). *Research on the Mechanism and Passive Control of Attached Cavitation on Hydrofoil*.

[51] Zhu Y., Li G., Wang R., Tang S., Su H., Cao K. (2021). Intelligent fault diagnosis of hydraulic piston pump combining improved lenet-5 and pso hyperparameter optimization. *Applied Acoustics*.

